# Mandibular response after rapid maxillary expansion in class II growing patients: a pilot randomized controlled trial

**DOI:** 10.1186/s40510-017-0189-6

**Published:** 2017-11-06

**Authors:** Roberta Lione, Valerio Brunelli, Lorenzo Franchi, Chiara Pavoni, Bernardo Quiroga Souki, Paola Cozza

**Affiliations:** 1 Department of Clinical Sciences and Translational Medicine, University of Rome “Tor Vergata”, Viale Oxford, 81, 00133 Rome, Italy; 2 Department of Dentistry UNSBC, Tirana, Albania; 3 Department of Surgery and Translational Medicine, University of Florence, Florence, Italy; 4 Thomas M. Graber Visiting Scholar, Department of Orthodontics and Pediatric Dentistry, School of Dentistry, The University of Michigan, Ann Arbor, MI USA; 5 School of Dentistry, Orthodontics, Pontifical Catholic University of Minas Gerais, Belo Horizonte, Brazil

**Keywords:** Rapid maxillary expansion, Mandibular response, Class II malocclusion, Growing subjects

## Abstract

**Background:**

The aim of this pilot randomized controlled trial (RCT) was to evaluate the sagittal mandibular response induced by rapid maxillary expansion (RME) therapy in mixed dentition patients with class II malocclusion, comparing the effects of bonded RME and banded RME with a matched untreated class II control group.

**Methods:**

This RCT was designed in parallel with an allocation ratio of 1:1:1. The sample consisted of 30 children with a mean age of 8.1 ± 0.6 years who were randomly assigned to three groups: group 1 treated with bonded RME, group 2 treated with banded RME, and group 3 the untreated control group. All patients met the following inclusion criteria: early mixed dentition, class II molar relationship, transverse discrepancy ≥ 4 mm, overjet ≥ 5 mm, and prepubertal skeletal maturity stage (CS1–CS2). The expansion screw was activated one quarter of a turn per day (0.25 mm) until overcorrection was reached. For each subject, lateral cephalograms and plaster casts were obtained before treatment (T1) and after 1 year (T2). A randomization list was created for the group assignment, with an allocation ratio of 1:1:1. The observer who performed all the measurements was blinded to group assignment. The study was single-blinded in regard to statistical analysis.

**Results:**

RME was effective in the correction of maxillary deficiency. Class II patients treated with both types of RME showed no significant improvement of the anteroposterior relationship of the maxilla and the mandible at both skeletal and occlusal levels. The acrylic splint RME had significant effects on reducing the skeletal vertical dimension and the gonial angle.

**Conclusions:**

The orthopedic expansion did not affect the sagittal relationship of class II patients treated in the early mixed dentition when compared with the untreated control group. Additional studies with a larger sample are warranted to elucidate individual variations in dento-skeletal mandibular response to the maxillary expansion protocol in class-II-growing patients.

**Trial registration:**

ClinicalTrials.gov NCT03159962.

## Background

Class II malocclusions are commonly observed in orthodontic patients [1]. During treatment planning among the several dento-skeletal pattern combinations of class II malocclusion, it is important to consider the maxillary transverse deficiency, which is often overlooked [2].

Tollaro et al. [3] showed an underlying posterior interarch transverse discrepancy of 3 to 5 mm in subjects in early mixed dentition with class II malocclusions without posterior crossbites in centric occlusion. When these class II patients are asked to posture their lower jaw forward in a class I molar relationship, this transverse discrepancy (i.e., maxillary constriction) can be observed clinically [3]. It was postulated that in these subjects, the mandible is kept in a distal position relative to centric relation because the constricted maxilla is holding it back [4, 5]. The presence of a primitive transverse discrepancy between the dental arches induces a backward position of the mandible, as the occlusal goal is to obtain the highest number of functional contacts [5].

As reported by several authors [6–9], widening the maxilla with rapid maxillary expansion often leads to spontaneous forward posturing of the mandible during the retention period. The orthopedic expansion removes occlusal interferences, allowing the mandible to posture forward, thus improving the sagittal relationships [10, 11]. The mandibular arch acts as a “foot” that moves forward after the “shoe” is widened [4, 5]. Caprioglio et al. reported that patients with smaller mandibular length and more acute superior gonial angles are expected to show greater improvement in class II molar relationship [2].

However, the effectiveness of rapid maxillary expansion (RME) on the sagittal dental or skeletal parameters is still controversial because very little has been written regarding the behavior of anteroposterior mandibular changes in class-II-growing subjects who underwent RME as the phase 1 treatment intervention. The reported significant occlusal improvement could be attributed to other reasons, i.e., skeletal growth or the use of additional appliances during the transition from mixed to permanent dentition. Moreover, the majority of the studies [3, 6–9] show some limits: they are not randomized [12], they are not prospective, and they have no control group or they use patients from growth studies as a source for the control group.

Considering that it was not possible to estimate from previous studies the standard deviation to be used for sample size calculation of the main trial with special regards to the type of intervention and observation intervals, the primary objective of the present investigation was to conduct a pilot randomized controlled trial (RCT) evaluating the changes in the anteroposterior mandibular position induced by bonded or banded RMEs compared with an untreated class II control group.

## Methods

The Consolidated Standards of Reporting Trials (CONSORT) checklist was used as a guideline for conducting and reporting this trial [13]. The present pilot RCT was designed as a prospective three-arm parallel group randomized clinical trial with a 1:1:1 allocation ratio.

The study was approved by the Ethics Committee at the University of XXXX, (protocol number 130/14), and informed consent was obtained from the patients’ parents. The trial was registered on ClinicalTrials.gov (registration number: NCT03159962).

A total of 30 subjects with a mean age of 8.1 ± 0.6 years (range 6.6–9.1 years) who sought for an orthodontic treatment, were enrolled in the Department of Orthodontics at the University of XXXX. All children met the following inclusion criteria: early mixed dentition with first molars fully erupted, class II malocclusion (full-cusp or end-to-end molar relationships), negative posterior transverse interarch discrepancy ≥ 4 mm [3], overjet ≥ 5 mm, and prepubertal stage of development (CS1–CS2 in cervical vertebral maturation) [14]. Exclusion criteria included previous orthodontic treatment, extracted or congenitally missing teeth, craniofacial syndromes or clefts, and use of additional orthodontic devices during the observation period.

**Fig. 1:**
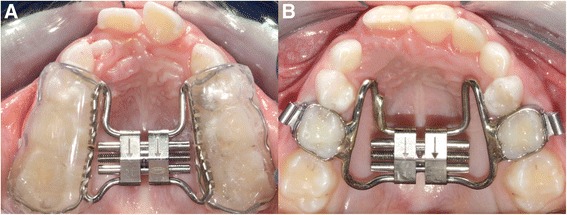
**a** Bonded RME. **b** Banded RME

Patients enrolled in the study were blindly assigned in three groups. In the first group (TG1), all subjects underwent a standardized treatment protocol with bonded RME with a 13-mm screw (A0620-13, Leone, Sesto Fiorentino, Firenze, Italy). The acrylic splints of the bonded expander extended from the first deciduous molars through the first permanent molars (Fig. 1a). In the second group (TG2), all children were treated with a banded RME in the form of a butterfly palatal expander with a 13-mm screw (A0620-13, Leone, Sesto Fiorentino, Firenze, Italy) [15] cemented through bands on the second deciduous upper molars (Fig. 1b), while subjects assigned to the third group served as the untreated control group (CG). Both TGs were consecutively treated by one clinician (R.L). The expansion screw was activated one quarter of a turn per day (0.25 mm per turn) until the palatal cusps of the maxillary posterior teeth approximated the lingual cusps of the mandibular posterior teeth. The expander was kept in place as a passive retainer for 8 months. After expander removal, patients were followed without performing any additional treatment for 4 months.

For each treated patient, standard lateral cephalometric radiographs were obtained before treatment (T1) and after 1 year (T2) to evaluate the T2–T1 dento-skeletal changes. The CG was followed up without treatment for 1 year and had lateral cephalograms before (T1) and after a 1-year interval (T2).

The cephalograms were scanned using a professional table scanner (Epson Perfection V700 Photo, CA, USA), with resolution set to 150 dots per inch (dpi) gray scale. Cephalograms were digitized by one investigator (V.B.). A customized digitization regimen and analysis (Viewbox 3.1; dHAL Software, Kifissia, Greece) were used for all the cephalograms that were examined in this study. All lateral cephalograms were at a magnification of 0%. The examiner who analyzed the lateral cephalograms of all children at T1 and T2 was blinded to the origin of the films and the group to which each subject belonged.

**Fig. 2:**
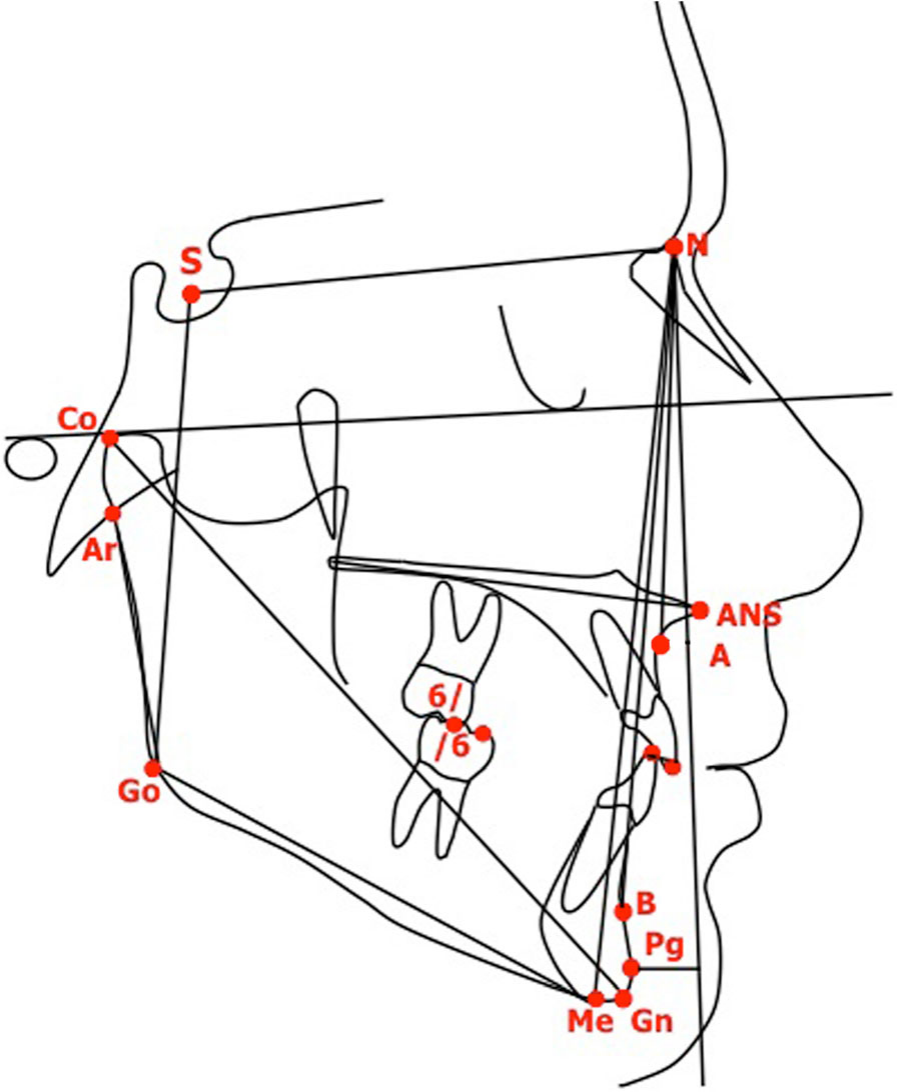
Cephalometric points, lines, and angles used in analysis: SNA angle (maxillary sagittal position), SNB angle (mandibular sagittal position), ANB angle (maxillomandibular sagittal discrepancy), point Pg to Nasion perpendicular (sagittal mandibular position relative to Frankfurt plane), mandibular total length (Co-Gn), SN to mandibular plane (Me-Go), gonial angle (Ar-Go-Me), lower anterior facial height (ANS-Me), overjet, overbite; molar relationship

The cephalometric reference points, lines, and angles used in the analysis are shown in Fig. 2.

The primary outcome was the change in the position of point Pogonion to the Nasion perpendicular (Pg to Nperp). Secondary outcomes were considered: (1) occlusal improvement of class II molar relationship and (2) treatment effects on a vertical dimension.

A sample size for this pilot trial was calculated according to the method proposed by Whitehead et al. [16]. For a standardized effect size of 1 (a clinically relevant change of 2.0 mm with a combined SD of 2.0 mm derived from Guest et al. [8]) for the primary outcome variable Pg to Nperp, a sample size of 10 subjects per group was required for a type I error rate of 5% and a power of 80%.

**Fig. 3:**
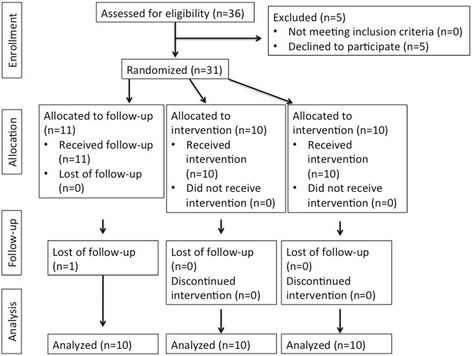
Study flow chart

Allocation of patients to the three groups was determined by a computer-generated randomization list using Rv.0.1 software [17] and by a block size of 4 (Fig. 3). Then, the allocation information (randomization results) was concealed in opaque and sealed envelopes by the statistician (C.C.).

The observer who performed all the measurements was blinded to the group assignment. The study was blinded in regard to the statistical analysis: blinding was obtained by eliminating from the elaboration file every reference to patient group assignment.

To determine the reliability of the method, 15 radiographs chosen at random were traced and digitized by the same investigator on two separate occasions at least 1 month apart. A paired *t* test was used to compare the two measurements (systematic error). The magnitude of the random error was calculated by using the method of moment’s estimator (MME) [18].

Exploratory statistics revealed that not all cephalometric variables were normally distributed (Kolmogorov-Smirnov test) with equality of variances (Levene’s test). Kruskal-Wallis test or ANOVA with Tukey’s post-hoc tests were used to compare the T2–T1 changes in the three groups. All changes were considered significant at *P* < 0.05.

All statistical computations were performed with SPSS software (Statistical Package for the Social Sciences, SPSS, Version 12, Chicago, IL, USA).

## Results

In this pilot trial, where 31 patients were randomly assigned to the interventions, one drop-out was observed in the CG. The final sample that received the intended treatment and analysis was 30 patients (Fig. 3). The recruitment started in December 2014 and the observation period ended in June 2016.

The baseline age was 8.1 ± 0.6 years (range 6.6–9.1 years). No significant between-group differences were found at T1 for any of the cephalometric variables (Table 1).

**Table 1:** Descriptive statistics and statistical comparisons of starting forms (ANOVA with Tukey’s post hoc tests)

Variables	RME bonded (1) (*n* = 10)		RME banded (2) (*n* = 10)		Controls (3) (*n* = 10)		*P*	1 vs 2	2 vs 3	1 vs 3
	Mean	SD	Mean	SD	Mean	SD				
Age	8.1	0.6	8.1	0.6	8.0	0.8	0.977	0.0	0.1	0.1
Sagittal skeletal										
SNA (deg)	79.2	3.5	81.3	2.6	82.2	3.7	0.147	− 2.1	− 0.9	− 3.0
SNB (deg)	74.4	3.6	76.2	2.8	76.4	3.8	0.196	− 1.8	− 0.2	− 2.0
ANB (deg)	4.6	1.2	5.1	1.2	5.8	1.5	0.152	− 0.5	− 0.7	− 1.2
Pg to NPerp (mm)	− 9.1	2.7	− 7.1	2.5	− 7.9	3.8	0.356	− 2.0	0.8	− 1.2
Co-Gn (mm)	93.8	3.5	91.3	4.2	93.5	5.8	0.442	2.5	− 2.2	0.3
Vertical skeletal										
SN to mandibular plane (deg)	33.8	4.3	33.6	5.6	30.7	5.2	0.334	0.2	2.9	3.1
ArGoMe (deg)	129.5	3.6	128.2	6.8	127.5	2.8	0.641	1.3	0.7	2.0
LAFH (mm)	51.6	2.9	51.6	3.0	51.3	3.1	0.972	0.0	0.3	0.3
Interdental										
Overjet (mm)	5.4	1.2	5.9	2.2	5.6	1.6	0.796	− 0.5	0.3	− 0.2
Overbite (mm)	0.6	0.9	0.7	1.9	1.8	1.3	0.124	− 0.1	− 1.1	− 1.2
Molar relationship (mm)	− 2.9	0.9	− 2.3	1.3	− 2.9	1.2	0.465	− 0.6	0.6	0.0

*deg* degrees

*P* < 0.05

No systematic error was found between the repeated cephalometric values. For the cephalometric variables, the random error varied from 0.21° (SNA angle) to 0.32° (gonial angle) for angular measurements and from 0.16 mm (Co-Gn) to 0.24 mm (overbite) for linear measurements.

As for the T2–T1 changes (Table 2), no statistical significant differences were pointed out for the sagittal position of the maxilla and the mandible at the end of the treatment with RME with respect to the untreated CG. The detectable significant changes occurred in TG1 with greater decreases of both facial divergency (TG1 vs TG2, − 1.1°; TG1 vs CG, − 1.5°) and gonial angle (TG1 vs TG2, − 1.3°; TG1 vs CG, − 1.5°). Treatment with RME did not affect significantly either dental measurements or molar relationship with respect to the untreated controls.

**Table 2:** Descriptive statistics and statistical comparisons on the T2–T1 changes (Kruskal-Wallis test or ANOVA or with Tukey’s post hoc tests)

Variables	RME bonded (1) (*n* = 10)		RME banded (2) (*n* = 10)		Controls (3) (*n* = 10)		*P*	1 vs 2	2 vs 3	1 vs 3
	Mean median	SD 25/75	Mean median	SD 25/75	Mean median	SD 25/75				
Age	1.0	0.1	1.0	1.0/1.1	1.0	0.1	0.742	0.0	0.0	0.0
Sagittal skeletal										
SNA (deg)	0.0	0.4	− 0.1	0.4	− 0.2	0.9	0.845	0.1	0.1	0.2
SNB (deg)	0.5	0.5	0.2	0.6	0.2	0.7	0.390	0.3	0	0.3
ANB (deg)	− 0.7	0.6	− 0.2	0.7	− 0.3	1.0	0.412	− 0.5	0.1	− 0.4
Pg to NPerp (mm)	0.8	0.7	0.4	1.0	0.1	1.2	0.296	0.4	0.3	0.7
Co-Gn (mm)	0.6	1.2	0.7	1.1	0.9	0.4	0.837	− 0.1	− 0.2	− 0.3
Vertical skeletal										
SN to mandibular plane (deg)	−0.6	0.8	0.5	0.6	0.9	0.7	0.000	− 1.1**	− 0.4	− 1.5***
ArGoMe (deg)	−0.9	0.7	0.4	1.2	0.6	1.3	0.012	− 1.3*	− 0.2	− 1.5*
LAFH (mm)	−0.3	0.8	0.5	0.7	0.6	1.1	0.064	− 0.8	− 0.1	− 0.9
Interdental										
Overjet (mm)	− 0.7	− 1.4/− 0.4	− 0.8	− 0.9/− 0.2	− 0.2	− 0.5/0.0	0.070	0.1	− 0.6	− 0.5
Overbite (mm)	0.5	0.3/2.2	0.2	− 0.1/1.5	0.3	− 0.4/0.9	0.154	0.3	− 0.1	0.2
Molar relationship (mm)	1.1	0.7	0.8	0.7	0.4	0.8	0.154	0.3	0.4	0.7

*deg* degrees, *25/75* 25th percentile/75th percentile

*P < 0.05; **P < 0.01; ***P < 0.001

## Discussion

Additional studies with a larger sample are warranted to elucidate individual variations in dento-skeletal mandibular response to the maxillary expansion protocol in class-II-growing subjects. However, the results of the present pilot RCT showed that there should be no concerns about the generalizability of results from a future definitive RCT conducted in an identical way to the pilot trial with a larger sample.

Transverse maxillary deficiency deserves to be included in the distinctive occlusal pattern of class II malocclusion [1–9]. Therefore, the aim of the present pilot RCT was to evaluate the sagittal mandibular response induced by RME therapy in mixed dentition patients with class II malocclusion, comparing the effects of bonded RME and banded RME with a matched untreated class II control group.

Several studies [10, 19, 20] investigated possible spontaneous correction of class II malocclusion after orthopedic maxillary expansion. Wendling et al. [10], Baratieri et al. [19], and Farronato et al. [20] reported for all class II patients a statistically significant decrease in ANB angle obtained during treatment as a result of a significant increase in SNB angle [10, 19, 20].

As suggested by McNamara [21], during the post-RME period, mandibular anterior displacement may be observed because of the overexpansion of the maxilla. Thus, the spontaneous correction of patients with a tendency toward a class II malocclusion cannot be expected during the active expansion period but rather during the retention period. For this reason, in the present study, the mean T2–T1 interval was 12 months.

On the contrary, Chung et al. [6] and Volk et al. [22] demonstrated that RME protocol did not predictably improve the occlusal relationship in class II prepubertal patients, not supporting the “foot in the shoe” theory.

The results reported in literature [7, 8, 19–26] are not only contradictory, but also frequently based on deficient methodology, or lack of clinical relevance. More solid scientific evidence based on reliable methods and proper study designs is still lacking to test whether dental correction or mandibular anterior shift and/or supplementary growth takes place after RME.

In order to have a better predictability of the effectiveness of any therapy, it is advisable to consider not only controlled groups, but also randomization [27].

Some authors [7, 8] reported significant occlusal sagittal improvements during the transition from mixed to permanent dentition. That might have helped in occlusal anteroposterior changes at the end of RME therapy. To skip this factor, only early mixed dentition patients were enrolled at the beginning of the study. The phase of dentition was stable during the whole observational period referring any outcomes to mandibular growth or anterior shift.

To our knowledge, the present study is the first pilot RCT to analyze specifically whether maxillary expansion spontaneously corrects or improves a class II malocclusion. In the present investigation, the treatment with RME did not affect significantly the anteroposterior skeletal mandibular response as well as the molar relationship with respect to the CG. The results indicated neither mandibular shift nor supplementary growth occurred after RME. Positive T2–T1 change greater than 1 mm for the value Pogonion to N-perpendicular was considered as a clinically significant mandibular advancement. Three patients treated with bonded RME showed favorable changes of sagittal mandibular position (Pg to NPerp > + 1 mm), compared with only 1 of the 10 patients treated with banded RME and with only 1 of the 10 untreated subjects. This difference was not significant (*P* = 0.405), according to Fisher’s exact probability test.

However, statistically significant changes were observed in the TG1 as a result of the acrylic splint, with a greater reduction of both facial divergency (TG1 vs TG2, − 1.1°; TG1 vs CG −1.5°) and gonial angle (TG1 vs TG2, − 1.3°; TG1 vs CG, − 1.5°) when comparing the TG1 both with the TG2 and the CG.

In bonded RME, the acrylic coverage of the occlusal surfaces, acting as a bite block, inhibited posterior dental extrusion and might provide some intrusion of posterior teeth [28]. Bonded RME is then suggested for correction of the transverse dimension in patients, who need to better control the vertical growth pattern minimizing tipping of the posterior maxillary teeth [23, 24, 29, 30]. Our results are in contrast with those reported by Guest et al. [8] and McNamara et al. [7]. In these large-scale investigations, the improvement in molar relationship in RME group were of over 1 mm in 92% of the expansion patients and over 2 mm in almost 50% of them without definitive class II mechanics incorporated into the protocol. When the authors compared the treated group with the historical control group, the net molar relationship improvement was 1.7 mm, mainly due to a significant increase in mandibular length. However, the mean observation interval in the previous studies was of 4 years, while in the present study all treated and untreated subjects were prospectively evaluated after 1 year.

## Conclusions

Class II patients in early mixed dentition treated with either bonded or banded RME showed no significant improvement of the anteroposterior relationship of the maxilla and the mandible at both skeletal and occlusal level when compared with an untreated control group. The treatment with bonded RME determined a reduction of the facial divergency and of the gonial angle when compared both with subjects treated with the banded RME and with untreated subjects.

The future definitive RCT will be planned with a larger sample without any changes from the pilot trial.
