# Correction to: Mandibular response after rapid maxillary expansion in class II growing patients: a pilot randomized controlled trial

**DOI:** 10.1186/s40510-018-0231-3

**Published:** 2018-07-12

**Authors:** Roberta Lione, Valerio Brunelli, Lorenzo Franchi, Chiara Pavoni, Bernardo Quiroga Souki, Paola Cozza

**Affiliations:** 1 Department of Clinical Sciences and Translational Medicine, University of Rome “Tor Vergata”, Viale Oxford, 81, 00133 Rome, Italy; 2 Department of Dentistry UNSBC, Tirana, Albania; 3 Department of Surgery and Translational Medicine, University of Florence, Florence, Italy; 4 Thomas M. Graber Visiting Scholar, Department of Orthodontics and Pediatric Dentistry, School of Dentistry, The University of Michigan, Ann Arbor, MI USA; 5 School of Dentistry, Orthodontics, Pontifical Catholic University of Minas Gerais, Belo Horizonte, Brazil

## Correction

In the publication of this article [1] there is an error in the Methods section. This has now been included in this correction. Instead of University of XXXX, it should instead read University of Rome “Tor Vergata”.

The error: “The study was approved by the Ethics Committee at the University of XXXX, (protocol number 130/14), and informed consent was obtained from the patients’ parents. The trial was registered on ClinicalTrials.gov (registration number: NCT03159962).

A total of 30 subjects with a mean age of 8.1 ± 0.6 years (range 6.6–9.1 years) who sought for an orthodontic treatment, were enrolled in the Department of Orthodontics at the University of XXXX.”

Should instead read: “The study was approved by the Ethics Committee at the University of Rome “Tor Vergata”, (protocol number 130/14), and informed consent was obtained from the patients’ parents. The trial was registered on ClinicalTrials.gov (registration number: NCT03159962).

A total of 30 subjects with a mean age of 8.1 ± 0.6 years (range 6.6–9.1 years) who sought for an orthodontic treatment, were enrolled in the Department of Orthodontics at the University of Rome “Tor Vergata”.”
